# Supplementary material for: Global within-species phylogenetics of sewage microbes suggest that local adaptation shapes geographical bacterial clustering
Source: Commun Biol. 2023 Jul 8;6:700. doi: 10.1038/s42003-023-05083-8 (PMC10329687; doi:10.1038/s42003-023-05083-8)
Supplement: Supplementary file 2 — Supplementary Information [file 42003_2023_5083_MOESM2_ESM.docx]

**Supplementary Information**

**Global within-species phylogenetics of sewage microbes suggest that local adaptation shapes geographical bacterial clustering**

**Authors**

Marie Louise Jespersen^1,2^, Patrick Munk^1^, Joachim Johansen^2,3^, Rolf Sommer Kaas^1^, Henry Webel^2^, Håkan Vigre^1^, Henrik Bjørn Nielsen^3^, Simon Rasmussen^2^, Frank M. Aarestrup^1^.

**Affiliations**

^1^National Food Institute, Technical University of Denmark, Kongens Lyngby, Denmark

^2^ Novo Nordisk Foundation Center for Protein Research, Faculty of Health and Medical Sciences, University of Copenhagen, Copenhagen, Denmark

^3^ Clinical-Microbiomics A/S, Copenhagen, Denmark

**Author List Footnotes**

Correspondence: Simon Rasmussen (simon.rasmussen@cpr.ku.dk) and Frank M. Aarestrup ([fmaa@food.dtu.dk](mailto:fmaa@food.dtu.dk))

**Supplementary Figure 1. Sample times**. Sampling times at the 239 different sampling sites. In total 757 sewage samples from 101 different countries were collected. Colours show the World Health Organization (WHO) region (legend on top).

**Supplementary Figure 2. Testing different species ANI thresholds.** We tested the species threshold of 95% ANI, by running the analysis with different species thresholds. By increasing the threshold (97.5%) we identified one cluster subset (C2sub) which could be tested, and four subsets that did not meet our requirements for PERMANOVA testing. This subset did not cluster significantly according to geography (Supplementary Data 2). When decreasing the species ANI threshold, we identified five MAG species that differed from the initially identified species. Of these five, three comprised an original MAG species plus one or more additional MAGs (C2+, C5+, and C30+), one was a merge of two original MAG species (C27C28) and the last one was a new MAG species, not seen in the original analysis (C42). All the trees contained a group of samples (corresponding to an original MAG species, in the cases of C2+, C5+, C30+, and C27C28) and one or more outliers. Geographical clustering was significant in one of these trees (C2+), in which most of the outliers were from the same region. Taken together, these results suggest that there are not many new MAG species to be found, even though the ANI thresholds were decreased. In most of the cases, where new MAG species were identified, the novelty was based on outliers, potentially from the collapse of different bacterial species, which is also likely the case for C27C28.

**Supplementary Figure 3. Strain mixing. a.** Heatmap of the ANI between the ten different Salmonella strains spiked into 50 different human gut samples from Nissen *et al.*^1^. Seven strains are very similar (ANI: 98.2% - 99.9%) to each other. **b.** ASTRAL species tree of the ten different Salmonella strain reference genomes. The seven strains with high ANI are highlighted in the red square. c. ASTRAL species tree of the NC MAGs found by binning with VAMB of the 50 different human gut samples. Tips are labelled with the VAMB bin name. d. ASTRAL species tree of the seven Salmonella strain reference genomes highlighted in b. In the trees in b, c, and d the tips are coloured according to the reference strain legend in b. ANI: Average Nucleotide Identity, NC: Near Complete, MAGs: Metagenome Assembled Genomes.

**Supplementary Figure 4. Phylogenetic binner trees.** ASTRAL species trees for the 32 MAG species that were included in the analysis. Tips are coloured to highlight the binning method for identification of each MAG. R^2^ values from PERMANOVA testing of ASTRAL species trees from the binning method of each MAG. Species trees were tested with PERMANOVA if they contained MAGs from both binners (MetaBAT and VAMB).

**Supplementary Figure 5. Phylogenetic trees.** ASTRAL species trees for the 33 MAG species that contained at least two samples from more than one region. The species trees are created from gene trees of orthologous genes identified in each MAG species, between 1,437 and 4,967 different genes were found for a species. Tips are coloured to highlight the WHO regional origin of each MAG (legend in bottom).

**Supplementary Figure 6. Outlier removal.** In five trees (C1, C2, C4, C14, and C24) with significant geographical clustering outliers were identified. In one case (C14), the tree consisted of two groups of MAGs and could not be tested, if one of the groups were removed. The four remaining trees were pruned from outliers and re-tested with PERMANOVA. In three cases (C1prun, C2prun, and C4prun) the geographical clustering was still significant after pruning of the trees (Supplementary Data 2). In the last case (C24prun) the tree was reduced to comprise five MAGs, because the initial tree consisted of two groups of MAGs. Taken together, 10 out of the 12 significant MAG species trees do not have outliers or are still significant after pruning, the last two trees are potentially comprising distinct subgroups within the species.

**

**Supplementary Figure 7. Regional clustering according to phyla.** Geographical R^2^ values from PERMANOVA testing of ASTRAL species trees from 33 MAG species divided into phylum groups. Species trees were tested with PERMANOVA if they contained at least two MAGS from at least two different regions. Additionally, if a region was represented by only one MAG in a tree, this tip was removed from the tree prior to testing. No significant difference was found in geographical clustering between different phyla (Kruskal-Wallis rank sum test, P: 0.70). The boxplot center, lower and upper hinge correspond to the median, first and third quantiles respectively. The upper and lower whiskers extend to the largest and smallest values, no more than 1.5* the inter-quartile range (IQR, ie. the distance between first and third quartiles). Data points beyond these values are plotted as individual outliers.

**

**Supplementary Figure 8. Gene variation according to gene groups.** Gene variation was calculated as mean pi, ie. the pairwise fraction of sites with differences. The plot shows log to mean pi stratified by gene groups for each MAG species. 28 of the 32 tested MAG species had significantly lower variance in organelle genes than in membrane genes, when testing for any significance between gene groups with a Kruskal-Wallis Rank Sum Test and subsequently testing for significance between specific gene groups with a Pairwise Wilcoxon Rank Sum test and after Benjamini-Hochberg correction of p values. Exact p values, test statistics, and 95% confidence intervals can be found in Supplementary Data 2. *: P<0.05, **: P<0.01, ***: P<0.001. The boxplot center, lower and upper hinge correspond to the median, first and third quantiles respectively. The upper and lower whiskers extend to the largest and smallest values, no more than 1.5* the inter-quartile range (IQR, ie. the distance between first and third quartiles). Data points beyond these values are plotted as individual outliers.

**

**Supplementary Figure 9. Geographical clustering in the different gene groups.** R^2^ values from PERMANOVA testing on gene trees stratified by gene groups. Nine of the 32 tested MAG species showed significantly lower R^2^ values for organelle genes than membrane genes. Statistical testing was performed as a Kruskal-Wallis test applied to all MAG species to test for any differences in R^2^ values between gene groups, followed by a Wilcoxon test on the MAG species with significance in the first test. The significance level highlighted in the plot is between organelle and membrane genes after Benjamini-Hochberg correction of Wilcox p values. Exact p values, test statistics, and 95% confidence intervals can be found in Supplementary Data 2. *: P<0.05, **: P<0.01, ***: P<0.001. The boxplot center, lower and upper hinge correspond to the median, first and third quantiles respectively. The upper and lower whiskers extend to the largest and smallest values, no more than 1.5* the inter-quartile range (IQR, ie. the distance between first and third quartiles). Data points beyond these values are plotted as individual outliers.

**

**Supplementary Figure 10. dN/dS in the different gene groups.** dN/dS values for the nine MAG species with significant differences in geographical R^2^ values between organelle and membrane genes. dN/dS were calculated with codeml using gene trees and multiple alignments as inputs. The plotted values are log to dN/dS. Six MAG species showed significant difference in dN/dS values of organelle and membrane genes, with five (C3, C5, C14, C34, and C38) having higher dN/dS values in membrane genes and one (C37) having lower. Statistical testing was performed with a Kruskal-Wallis test to all nine MAG species, and subsequent Wilcox test on species displaying significance in the Kruskal-Wallis test. The significance level shown in the plot is the wilcox p-value between organelle and membrane genes after Benjamini-Hochberg correction. Exact p values, test statistics, and 95% confidence intervals can be found in Supplementary Data 2. *: P<0.05, **: P<0.01, ***: P<0.001. The boxplot center, lower and upper hinge correspond to the median, first and third quantiles respectively. The upper and lower whiskers extend to the largest and smallest values, no more than 1.5* the inter-quartile range (IQR, ie. the distance between first and third quartiles). Data points beyond these values are plotted as individual outliers.

*
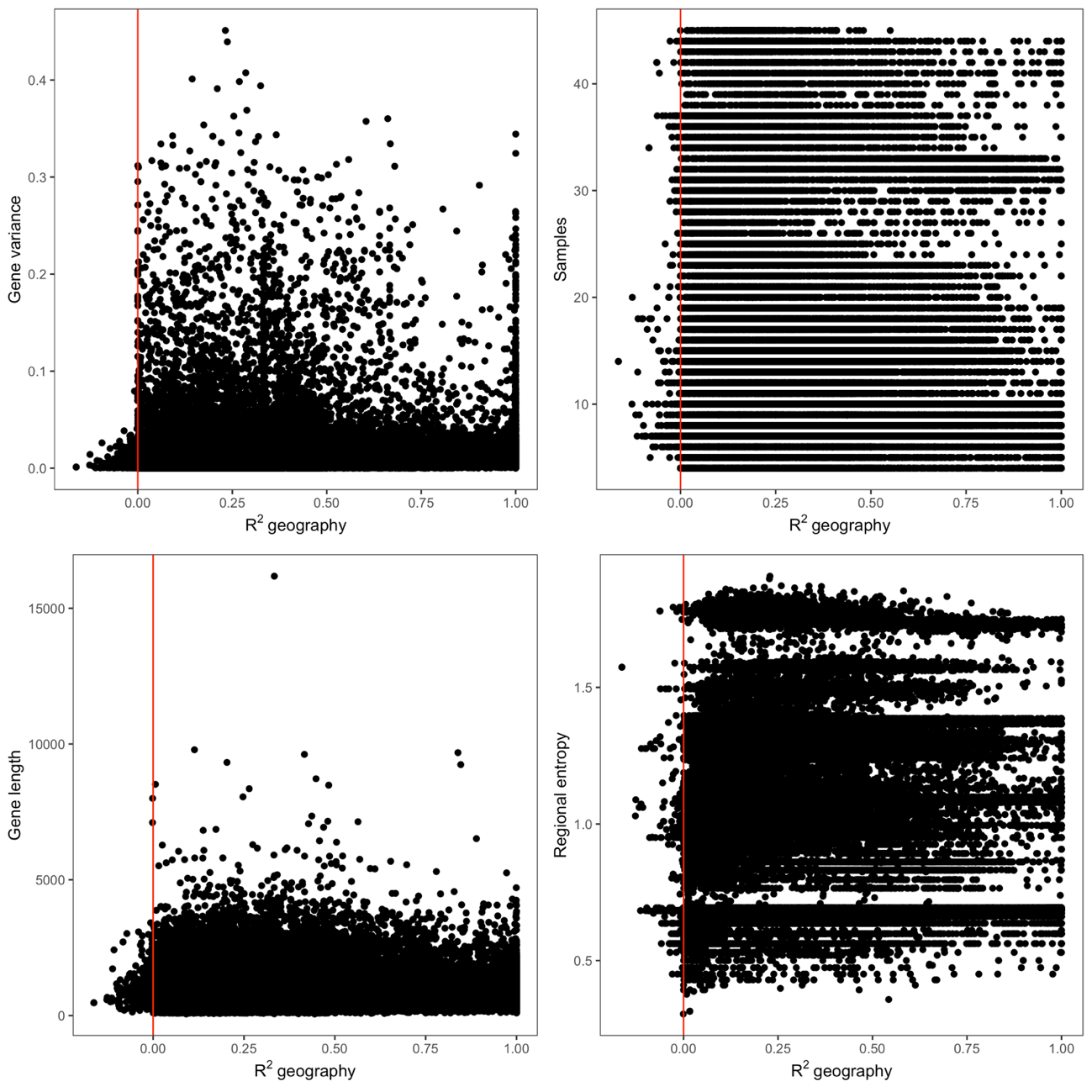
*

**Supplementary Figure 11. R^2^ values according to different gene- and tree variables.** Geographical R^2^ values from PERMANOVA testing of all gene trees of all tested MAG species. Tips of trees were removed prior to testing if being the only tip representing a region. Furthermore, trees were only tested if containing a least two regions each represented by more than one sample. The number of samples were counted from the gene trees after this removal of tips. Gene variance was calculated as mean pi (the mean fraction of varying sites between all pairwise sequences). Gene lengths were identified from fasta files and regional entropy was calculated based on the total number of region and the proportion of samples belonging to a specific region. Red lines indicate R^2^ values of zero. Some gene trees representing short genes with low variance got negative R^2^ values from the PERMANOVA test, these genes were excluded from further analysis of R^2^ values.

| **Sample** | **No. Salmonella** | **No. MAGs** | **ANI** | **MAG** | **Genome** | **Completeness** | **Contamination** | **dRep cluster** |
| --- | --- | --- | --- | --- | --- | --- | --- | --- |
| S11 | 2 | 2 | 98.9% | S11C354 | NC_021902 | 99.02 | 0.17 | 2_2 |
| S15 | 3 | 3 | 98.2%, 98.2%, 98.8% | S15C698 | NC_016810 | 97.08 | 0.72 | 2_2 |
| S16 | 2 | 2 | 98.7% | S16C698 | NC_016810 | 97.58 | 1.67 | 2_2 |
| S17 | 1 | 1 | NA | S17C263 | NC_022221 | 99.88 | 0.04 | 2_2 |
| S19 | 1 | 1 | NA | S19C698 | NC_003197 | 100.00 | 0.04 | 2_2 |
| S21 | 1 | 1 | NA | S21C3667 | NC_010067 | 99.85 | 0.04 | 2_1 |
| S25 | 2 | 2 | 98.7% | S25C698 | NC_016810 | 94.85 | 2.02 | 2_2 |
| S27 | 1 | 1 | NA | S27C698 | NC_022544 | 100.00 | 0.04 | 2_2 |
| S28 | 1 | 1 | NA | S28C751 | NC_011094 | 100.00 | 0.16 | 2_2 |
| S29 | 3 | 3 | 98.2%, 98.2%, 98.8% | S29C354 | NC_021902 | 95.09 | 1.24 | 2_2 |
| S2 | 1 | 1 | NA | S2C3667 | NC_010067 | 99.85 | 0.04 | 2_1 |
| S30 | 1 | 1 | NA | S30C3667 | NC_010067 | 99.85 | 0.04 | 2_1 |
| S31 | 2 | 2 | 98.7 % | S31C354 | NC_021902 | 98.52 | 1.56 | 2_2 |
| S38 | 2 | 1 | 99.9% | S38C698 | NC_003197 | 100.00 | 0.33 | 2_2 |
| S39 | 1 | 1 | NA | S39C698 | NC_003197 | 100.00 | 0.04 | 2_2 |
| S42 | 1 | 1 | NA | S42C698 | NC_003197 | 100.00 | 0.04 | 2_2 |
| S45 | 2 | 2 | 98.9% | S45C698 | NC_022544 | 96.90 | 2.56 | 2_2 |
| S46 | 1 | 1 | NA | S46C354 | NC_021902 | 100.00 | 0.04 | 2_2 |
| S47 | 2 | 1 | 99.9% | S47C698 | NC_003197 | 99.34 | 1.50 | 2_2 |
| S49 | 1 | 1 | NA | S49C354 | NC_021902 | 100.00 | 0.04 | 2_2 |
| S4 | 2 | 1 | 99.9% | S4C698 | NC_022544 | 100.00 | 0.04 | 2_2 |
| S5 | 2 | 1 | 99.9% | S5C698 | NC_003197 | 100.00 | 0.04 | 2_2 |
| S6 | 1 | 1 | NA | S6C354 | NC_021902 | 100.00 | 0.04 | 2_2 |
| S8 | 1 | 1 | NA | S8C3667 | NC_010067 | 99.85 | 0.04 | 2_1 |
| S9 | 1 | 1 | NA | S9C751 | NC_011094 | 100.00 | 0.16 | 2_2 |

**Supplementary Table 1. Strain mixing.** Overview of NC MAGs from the strain mixing experiment. No. Salmonella lists the number of Salmonella strains that were spiked into a given sample and no. bins shows the number of MAGs identified with VAMB in each sample. The ANI column shows the ANI between the different strains spiked into the samples. MAG and genome list the name of the VAMB MAGs and the Salmonella strain genomes they were identified as in Nissen *et al.* 2021. Completeness and Contamination are from CheckM quality control of the MAGs and dRep cluster lists the cluster number from dRep comparisons. We identified 25 NC MAGs from 25 different samples which were clustered into two different MAG species with dRep, one of these contained 21 MAGs (dRep cluster 2_2). Of these 21 MAGs only four were identified in samples where the number of VAMB MAGs identified was not the same as the number of spiked Salmonella strains. In all these four cases the ANI between the Salmonella strains were very high (99.9%). The data in the columns No. Salmonella, No. MAGs, MAG, and Genome are from Nissen *et al.*^1^. ANI: Average Nucleotide Identity, NC: Near Complete, MAGs: Metagenome Assembled Genomes.

| **MAG species** | **Gene variance** | **Gene length** | **Number of samples** | **Regional entropy** |
| --- | --- | --- | --- | --- |
| C1 | 0,013 | 0,024 | 0,046 | 0,064 |
| C2 | -0,016 | 0,098 | 0,295 | 0,247 |
| C3 | 0,103 | -0,06 | -0,283 | -0,208 |
| C4 | 0,068 | -0,032 | -0,182 | -0,004 |
| C5 | 0,224 | 0,085 | -0,119 | -0,05 |
| C6 | 0,093 | -0,114 | -0,273 | -0,128 |
| C7 | 0,115 | 0,031 | -0,146 | -0,019 |
| C8 | 0,076 | 0,136 | -0,077 | 0,005 |
| C9 | 0,095 | -0,028 | -0,132 | -0,041 |
| C11 | 0,099 | 0,051 | -0,189 | -0,132 |
| C12 | -0,032 | 0,184 | 0,005 | 0,023 |
| C13 | 0,139 | 0,057 | -0,201 | -0,108 |
| C14 | 0,003 | 0,155 | 0,114 | 0,038 |
| C15 | 0,069 | -0,003 | -0,064 | -0,024 |
| C16 | -0,133 | -0,045 | 0,288 | 0,324 |
| C17 | 0,049 | 0,021 | 0,063 | 0,122 |
| C18 | 0,071 | 0,004 | -0,075 | 0,07 |
| C20 | -0,051 | -0,023 | 0,076 | 0,126 |
| C21 | 0,047 | 0,022 | -0,016 | 0,018 |
| C22 | 0,022 | 0,037 | 0,079 | 0,192 |
| C23 | 0,119 | -0,017 | -0,17 | 0,164 |
| C24 | 0,022 | 0,076 | 0,075 | 0,141 |
| C25 | 0,039 | 0,005 | -0,185 | 0,009 |
| C26 | -0,38 | -0,001 | 0,486 | 0,432 |
| C28 | -0,011 | 0,03 | 0,067 | 0,075 |
| C29 | 0,061 | 0,051 | -0,096 | 0,042 |
| C30 | 0,062 | -0,036 | -0,037 | 0,112 |
| C31 | 0,012 | 0,075 | -0,035 | 0,031 |
| C32 | -0,004 | 0,016 | 0,163 | 0,216 |
| C34 | 0,113 | 0,074 | -0,139 | 0,076 |
| C37 | -0,018 | 0,03 | 0,123 | 0,123 |
| C38 | 0,294 | 0,179 | 0,058 | 0,058 |
| C41 | 0,015 | 0,034 | NA | NA |

**Supplementary Table 2. R2 correlations.** R2 values from PERMANOVA testing of gene trees were tested for correlation with gene variance, gene length, number of samples and regional entropy by calculating the Pearson Correlation Coeeficient (PCC). Gene variance was calculated as mean fraction of sites with variation across all pairwise sequences. Gene lengths were counted as number of bases from multiple fasta files of orthologous genes. Number of samples were counted as tips in the gene trees after removal of samples according to trimming prior to PERMANOVA testing. Regional entropy was calculated using proportion of samples belonging to a certain region and the total number of regions in a tree. The PCC for each gene tree variable for all MAG species is shown in the table. In one MAG species (C26) we found a correlation (PCC>0.4 or PCC<-0.4) between the number of samples and the regional entropy and the geographical R2 values of gene trees. This MAG species was excluded from further analysis of gene group R2 values. For one MAG species (C41) the number of samples and the regional entropy were the same in all gene trees and the PCC could therefore not be calculated (Standard deviation = 0).

**Supplementary Data**

Supplementary Data 1 (excel file). Taxonomy

Supplementary Data 2 (excel file). P-values and test statistics

Supplementary Data 3 (excel file). Sample list

**Supplementary References**

1. Nissen, J. N. *et al.* Improved metagenome binning and assembly using deep variational autoencoders. *Nat. Biotechnol.* (2021) doi:10.1038/s41587-020-00777-4.
