# Supplementary material for: Global within-species phylogenetics of sewage microbes suggest that local adaptation shapes geographical bacterial clustering
Source: Commun Biol. 2023 Jul 8;6:700. doi: 10.1038/s42003-023-05083-8 (PMC10329687; doi:10.1038/s42003-023-05083-8)
Supplement: Supplementary file 3 — Description of Additional Supplementary Files [file 42003_2023_5083_MOESM3_ESM.pdf]

Description of Additional Supplementary Files

File name: Supplementary Data 1

Description: Taxonomy

File name: Supplementary Data 2

Description: P-values and test statistics

File name: Supplementary Data 3

Description: Sample list
