# Supplementary material for: Global within-species phylogenetics of sewage microbes suggest that local adaptation shapes geographical bacterial clustering
Source: Commun Biol. 2023 Jul 8;6:700. doi: 10.1038/s42003-023-05083-8 (PMC10329687; doi:10.1038/s42003-023-05083-8)
Supplement: Supplementary file 7 — Reporting Summary [file 42003_2023_5083_MOESM7_ESM.pdf]

nature portfolio

Corresponding author(s): Simon Rasmussen and Frank M. Aarestrup

Last updated by author(s): Jun 14, 2023

Reporting Summary

Nature Portfolio wishes to improve the reproducibility of the work that we publish. This form provides structure for consistency and transparency in reporting. For further information on Nature Portfolio policies, see our Editorial Policies and the Editorial Policy Checklist.

Statistics

For all statistical analyses, confirm that the following items are present in the figure legend, table legend, main text, or Methods section.

n/a	Confirmed	
☐	☒	The exact sample size (*n*) for each experimental group/condition, given as a discrete number and unit of measurement
☐	☒	A statement on whether measurements were taken from distinct samples or whether the same sample was measured repeatedly
☐	☒	The statistical test(s) used AND whether they are one- or two-sided<br>*Only common tests should be described solely by name; describe more complex techniques in the Methods section.*
☐	☒	A description of all covariates tested
☐	☒	A description of any assumptions or corrections, such as tests of normality and adjustment for multiple comparisons
☐	☒	A full description of the statistical parameters including central tendency (e.g. means) or other basic estimates (e.g. regression coefficient) AND variation (e.g. standard deviation) or associated estimates of uncertainty (e.g. confidence intervals)
☐	☒	For null hypothesis testing, the test statistic (e.g. *F*, *t*, *r*) with confidence intervals, effect sizes, degrees of freedom and *P* value noted<br>*Give P values as exact values whenever suitable.*
☒	☐	For Bayesian analysis, information on the choice of priors and Markov chain Monte Carlo settings
☒	☐	For hierarchical and complex designs, identification of the appropriate level for tests and full reporting of outcomes
☒	☐	Estimates of effect sizes (e.g. Cohen's *d*, Pearson's *r*), indicating how they were calculated

Our web collection on statistics for biologists contains articles on many of the points above.

Software and code

Policy information about availability of computer code

Data collection	No code were used to collect data
Data analysis	metaSpades (v3.13)<br>MetaBAT2 (v2.10.2)<br>VAMB (v3.0.1)<br>Minimap2 (v2.6)<br>CheckM (v1.1.3)<br>dRep (v2.2.3)<br>GTDB-Tk (v0.3.2)<br>CoverM (v 0.6.1)<br>MASH (v2.0)<br>FastTree (v2.1.11)<br>iTol (v1.0)<br>Prodigal (v2.6.3)<br>Sonicparanoid (v1.3.4)<br>MAFFT (v7.453)<br>TrimAl (v1.4)<br>IQ-TREE (v1.6.8)<br>ASTRAL (v5.7.4)<br>ggtree package in R (v2.0.4)<br>InterProScan (v5.36-75.0)<br>GO.db package in R (v2.1)<br>paml (v.4.9j)

March 2021

CSI phylogeny (v1.4)
vegan package in R (v2.5-6)

For manuscripts utilizing custom algorithms or software that are central to the research but not yet described in published literature, software must be made available to editors and reviewers. We strongly encourage code deposition in a community repository (e.g. GitHub). See the Nature Portfolio guidelines for submitting code & software for further information.

Data

Policy information about availability of data

All manuscripts must include a data availability statement. This statement should provide the following information, where applicable:

- Accession codes, unique identifiers, or web links for publicly available datasets
- A description of any restrictions on data availability
- For clinical datasets or third party data, please ensure that the statement adheres to our policy

The raw reads are available in the European Nucleotide Archive (ENA) under the accession numbers: PRJEB40798, PRJEB40816, PRJEB40815, PRJEB27621, and ERP015409.

Field-specific reporting

Please select the one below that is the best fit for your research. If you are not sure, read the appropriate sections before making your selection.

☐ Life sciences ☐ Behavioural & social sciences ☒ Ecological, evolutionary & environmental sciences

For a reference copy of the document with all sections, see nature.com/documents/nr-reporting-summary-flat.pdf

Ecological, evolutionary & environmental sciences study design

All studies must disclose on these points even when the disclosure is negative.

Study description	The microbiomes of globally distributed sewage samples were investigated through metagenomics binning of bacterial genomes.
Research sample	The samples used in this study are from the Global Sewage Project. Samples in this manuscript include sewage collected from 241 sites and 101 different countries comprising 757 samples in total. The collection of these samples have been described in Hendriksen, R. S. et al. Global monitoring of antimicrobial resistance based on metagenomics analyses of urban sewage. Nat. Commun. 10, 1124 (03 08, 2019).
Sampling strategy	Sampling strategy is described in Hendriksen, R. S. et al. Global monitoring of antimicrobial resistance based on metagenomics analyses of urban sewage. Nat. Commun. 10, 1124 (03 08, 2019).
Data collection	Sample collection is described in Hendriksen, R. S. et al. Global monitoring of antimicrobial resistance based on metagenomics analyses of urban sewage. Nat. Commun. 10, 1124 (03 08, 2019).
Timing and spatial scale	The samples in this manuscript include samples from the Pilot project of the Global Sewage project performed in 2016 and subsequent sampling approximately every six months from 2017 untill 2019.
Data exclusions	Some samples initially thought to be included in the analysis, were excluded when it was found that these were not from sewage treatment plants but in stead from a river, hospital, or slaughter house, ie. these samples were not comparable to the remaining samples in the data set.
Reproducibility	The phylogenetic species trees created with ASTRAL were also created with another method (CSI phylogeny) giving similar results.
Randomization	Samples were grouped and tested based on their WHO region grouping. Identical testing was performed with continent grouping giving similar results for the species trees.
Blinding	Sample collection is described in Hendriksen, R. S. et al. Global monitoring of antimicrobial resistance based on metagenomics analyses of urban sewage. Nat. Commun. 10, 1124 (03 08, 2019).

Did the study involve field work? ☐ Yes ☒ No

Reporting for specific materials, systems and methods

We require information from authors about some types of materials, experimental systems and methods used in many studies. Here, indicate whether each material, system or method listed is relevant to your study. If you are not sure if a list item applies to your research, read the appropriate section before selecting a response.

Materials & experimental systems

n/a	Involved in the study
☒	☐ Antibodies
☒	☐ Eukaryotic cell lines
☒	☐ Palaeontology and archaeology
☒	☐ Animals and other organisms
☒	☐ Human research participants
☒	☐ Clinical data
☒	☐ Dual use research of concern

Methods

n/a	Involved in the study
☒	☐ ChIP-seq
☒	☐ Flow cytometry
☒	☐ MRI-based neuroimaging
